# Imaging appearance of a post-intubation cricoid chondronecrosis

**DOI:** 10.1259/bjrcr.20150442

**Published:** 2016-07-28

**Authors:** Kavitha Palled, Venkatraman Bhat, Suhel Hassan, Maneesh Ganamukhi

**Affiliations:** ^1^Department of Otolaryngology, Narayana Health, Shaw Mazumdar Cancer Center, Bengaluru, India; ^2^Department of Radiology, Narayana Health, Shaw Mazumdar Cancer Center, Bengaluru, India

## Abstract

Chondronecrosis is a known complication of external beam radiation therapy and endotracheal intubation. Radiation therapy is the most common cause of chondronecrosis owing to local cartilage ischaemia following treatment. Prolonged endotracheal intubation can lead to chondronecrosis as it is associated with excessive pressure on the cartilage by the endotracheal tube or its cuff. The cricoid ring is the most commonly affected cartilage. CT imaging is an integral part of the workup, although reports on imaging appearances are scant. We report the imaging and clinical presentation of a case of chondronecrosis secondary to the use of endotracheal tube ventilation. The patient was managed conservatively with good clinical outcome.

## Case report

**Figure 1.**
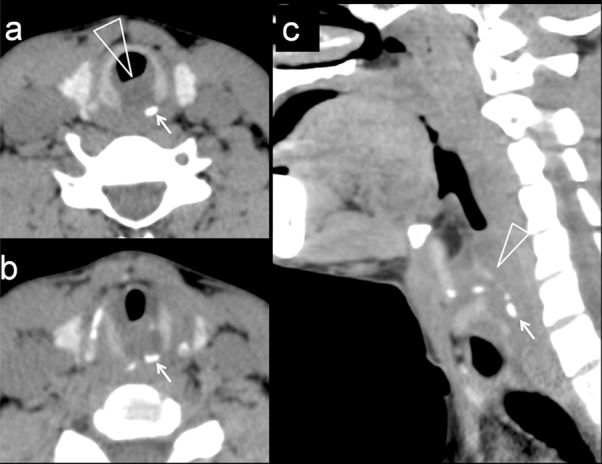
Axial non-contrast-enhanced CT image at the level of the cricoid cartilage (a) shows a discontinuous posterior ring with an isolated sclerotic component (arrow). There is diffuse soft tissue swelling, encroaching on the air column (triangle). (b) An additional axial image shows bone fragments (arrow). (c) Sagittal reconstruction demonstrates a hypodense postcricoid soft tissue swelling (triangle) with multiple bone fragments (arrow).

**Figure 2.**
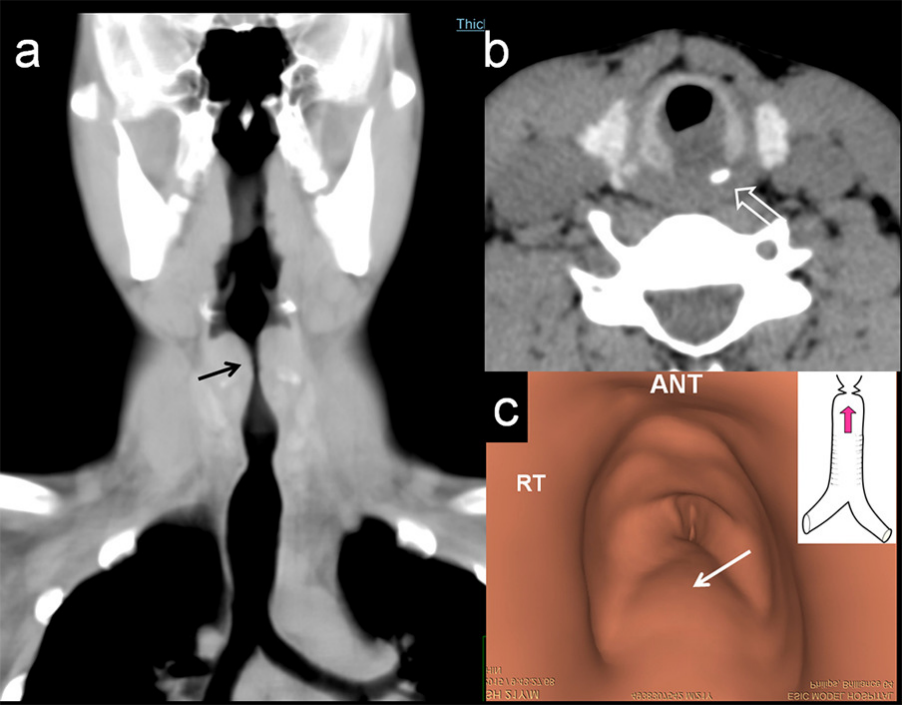
Coronal minimal intensity projection (a) shows a diffuse soft tissue swelling (black arrow) centred around the posterior arch of the cricoid cartilage with reduction in the airway calibre. (b) Axial CT image showing the cricoid bone fragment with the soft tissue swelling (open arrow). (c) Endoscopic view of the subglottic region from volumetric CT data, viewed caudocranially towards the subglottic region (inset) shows diffuse soft tissue swelling along the posterior wall of the subglottic trachea (arrow).

A-21-year-old-male patient presented with hoarseness of voice and occasional difficulty in breathing. The patient was involved in a road traffic accident 9 months ago and had sustained a head injury. He underwent surgery for depressed fracture of the parietal bone. The patient was managed on endotracheal intubation for 7 days, followed by elective tracheostomy. Later, he was put on T-piece mode of ventilation. Subsequently, he was decannulated successfully after 4 months. 10 days following decannulation, the patient developed change of voice—hoarse in nature and breathy in quality—chronic cough and difficulty in breathing. He was conservatively treated and later referred to our centre for further management. Video laryngoscopy showed a phonatory gap owing to restricted adduction of both vocal cords. Inflammatory markers were negative. Gastrointestinal endoscopy was normal. Multidetector CT (MDCT) scan showed soft tissue density in the cricopharyngeal region, which was encroaching on the tracheal air column from the posterior aspect. The cricoid ring was incomplete, fragmented with sclerotic components within the soft tissue density (Figure 1). The extent of narrowing of the air column and soft tissue bulge along the posterior wall of the subglottic trachea was well illustrated with a coronal image and a surface-rendered three-dimensional reconstruction (Figure 2). Voice recording showed severe hoarse voice with pitch breaks and a maximum phonatory duration of 6 s. Finally, based on clinical and imaging information, a diagnosis of cricoid chondronecrosis following prolonged intubation was considered. The patient was managed conservatively with steroids, physiotherapy and nebulization.

## Discussion

Laryngeal chondronecrosis is a known but relatively rare complication of endotracheal intubation. It is a well-known entity after radiation therapy. Radiation can compromise the blood supply to the soft tissues and cartilage of the larynx.^1,2^ The resultant ischaemia initiates perichondritis, leading to necrosis and sequestration of cartilage, if the integrity of the cartilage or its perichondrium is disrupted (*e.g.* owing to repeated intubation). This process may be accelerated by bacterial superinfection.^3^ Cricoid injury has been reported as a complication of endotracheal and nasogastric intubation. It is usually an acute injury secondary to ulceration of the retrocricoid mucosa, leading to perichondritis and chondritis, and ultimately leading to cricoid necrosis.^4–6^ Generally, normal remodelling of bone occurs in an orderly sequence. Initially osteoclasts are activated, presumably by abnormal pressure on the bone, leading to resorption of both organic and inorganic components before becoming quiescent.^6^ The gradual destruction of bone stimulates proliferation of new vessels, which invade the remaining bone. Osteoblasts form new bone and maintain the remodelled cortical margin.^4^ A combination of these events is the most likely explanation for the bone fragmentation and remodelling of cartilage noted in our patient. There are few reports on imaging appearance of cricoid chondronecrosis.^5–7^ Charlin et al^7^ reported an initial CT appearance demonstrating a low attenuation mass replacing the cartilage in a case of cricoid injury secondary to intubation. Subsequently, there was resolution of the mass, with residual demineralization of the cricoid.

Clinical disability in the form of voice changes and respiratory distress are graded by Chandler^2^ in patients with osteonecrosis secondary to radiation. Although the mechanism of necrosis is different, symptoms appear to follow a similar pattern. Our patient showed severity of Grade 2 symptoms and did not have odynophagia, as observed in Chandler's series of patients. Breathing difficulty can be attributed to the oedema around the subglottic trachea. CT scan abnormalities described in cricoid chondronecrosis include disruption of the cartilage ring, chondrolysis, sclerosis of the cartilage, fragmentation, distortion of anatomy and soft tissue swelling. Changes observed are non-specific and sometimes overlap with imaging features of an infective or a neoplastic process. Clinical context, laboratory reports and available histological details help in excluding those conditions. Imaging features may be the only objective evidence of abnormality when input from clinical examination is non-specific, as in our case. MDCT imaging plays a pivotal role in the evaluation of the larynx. Interpretation of cartilage anatomy may be challenging owing to a wide range of normal variations. MDCT provides precise information on the structural details of the cartilage and changes in soft tissues and airway. Demonstration of the extent of soft tissue component and airway encroachment is facilitated by multiplanar reformations and viewing in minimal intensity projection. Three-dimensional volumetric rendering is extremely valuable in showing mass effect or narrowing of the subglottic airway, especially if the glottic chink is narrow, precluding direct endoscopic vision. Endotracheal intubation-related chondronecrosis can be managed with steroids, nebulization and physiotherapy. Few patients may need total laryngectomy or permanent tracheostomy. However, majority of the patients are managed conservatively.

## Conclusions

Prompt recognition of laryngeal chondronecrosis is important to ensure effective management. MDCT imaging is the most important imaging tool for evaluation. Imaging observations are non-specific and cartilage changes resulting from these benign processes could be confused with the destructive changes secondary to inflammatory or malignant lesions. Clinical context and pathological data are helpful in differentiation. Endotracheal tube-induced chondronecrosis can be managed with steroids, nebulization and physiotherapy. Most cases can be managed without resorting to total laryngectomy or permanent tracheostomy.

## Learning points

1. Cricoid chondronecrosis is a known, relatively rare complication of prolonged intubation.
2. Imaging appearance of the cricoid chondronecrosis is infrequently described, although CT imaging constitutes an essential part of management.
3. CT scan findings include discontinuity of the cricoid arch, distortion of cartilage outline, chodrolysis, sclerosis, fragmentation and soft tissue swelling.
4. Lesion demonstration using multiple imaging planes, minimal intensity projection display and volume-rendered techniques can greatly complement in confirming the diagnosis.

## Consent

Informed patient consent obtained for publication.

## References

[1] Cukurova I, Cetinkaya EA Radionecrosis of the larynx: case report and review of the literature. Acta Otorhinolaryngologica Ital 2010; 30: 205–8. PMC3008149 21253286

[2] Chandler JR Radiation fibrosis and necrosis of the larynx. Ann Otol Rhinol Laryngol 1979; 88: 509–14. 475247 10.1177/000348947908800410

[3] Keene M, Harwood AR, Bryce DP, van Nostrand AW Histopathological study of radionecrosis in laryngeal carcinoma. Laryngoscope 1982; 92: 173–80. 7162313 10.1002/lary.1982.92.2.173

[4] Weissman JL, Curtin HD Benign erosion of laryngeal cartilage: report of two cases. AJNR Am J Neuroradiol 1990; 11: 1215–16. 2124063 PMC8332113

[5] Ali AA, Shweihat YR, Bartter T Cricoid chondronecrosis: a complication of endotracheal intubation. J Ark Med Soc 2012; 108: 192–4. 22435316

[6] Wiel E, Vilette B, Solanet C, Darras JA, Scherpereel P Chondronecrosis of the cricoid cartilage after intubation. Two case reports. Eur J Anaesthesiol Suppl 1997; 14: 461–3. 10.1046/j.1365-2346.1997.00116.x 9253578

[7] Charlin B, Dehon A, Bergeron D, Mongeau CJ, Grondin P Aseptic necrosis of the cricoid: a complication of tracheal intubation. J Otolaryngol 1987; 16: 377–81. 3694746

